# Nervous Diseases and Their Diagnosis

**Published:** 1887-04

**Authors:** 


NERVOUS DISEASES AND THEIR DIAGNOSIS. A treatise upon the phenomena, produced by diseases of the nervous system, with especial reference to the recognition of their causes. By H. C. Wood, M. D., LL.D., member of the National Academy of Science, Philadelphia. J. B. Lippincott Company. 1887.

It would seem that the author of this work did not count so much upon being a member of the medical staff of the University of Pennsylvania as on his scientific associations; or perhaps the idea of evolving a new method of presenting diseases of the nervous system, by which he cuts loose from the routine of medical schools, reconciled him to ignoring his professorial connection with that institution on his title-page.

Whether readers who desire systematic views of the relations of the different portions of the voluntary and ganglionic nerves, with their respective derangements, as connected with their centres, shall derive any advantage from this synthetic arrangement, is extremely problematical. It would seem that a course observed in studying a nervous disease originally by tracing peripheral disturbances to central lesions should then lead to the most satisfactory results in imparting this knowledge to others, but after grouping the data, the analytic process ought to prove the most effective mode of proceeding. This diagnostic neurology —as the author designates the undertaking—in the running general caption of the pages, treats in eleven chapters of the various deviation from the normal performance of the functions of the nervous system. While it is most assuredly a valuable collection of data connected with nervous derangements, and in so far an important addition to the history of diseases of the nervous system, yet, the writer fails to discover any well-defined method in the classification of disorders of the nerves or clearly drawn lines of distinction between them. The author, it is true, has presented facts which, by the inductive process, leads to knowledge, and has followed Hunter in his mode of demonstration; but the claim to point out the great landmarks of diagnosis calls for an analysis and distribution of the data which should be available in recognizing nervous diseases. The reader will search in vain for this clear distinction between allied nervous disorders, and in so far the term "diagnostic neurology" is a misnomer.

The introduction treats in a general way of that much-abused condition, neurasthenia; and then come paralysis, motor excitements, reflexes, disturbances of equilibration, trophic lesions, sensory paralysis, exaltations of sensibility, disturbances of the special senses, disorders of memory and consciousness, disorders of consciousness and disturbances of intellection. The book will well repay a perusal by practitioners.

